# Curcumin Attenuates Acute Graft-versus-Host Disease Severity via *In Vivo* Regulations on Th1, Th17 and Regulatory T Cells

**DOI:** 10.1371/journal.pone.0067171

**Published:** 2013-06-20

**Authors:** Min-Jung Park, Su-Jin Moon, Sung-Hee Lee, Eun-Ji Yang, Jun-Ki Min, Seok-Goo Cho, Chul-Woo Yang, Sung-Hwan Park, Ho-Youn Kim, Mi-La Cho

**Affiliations:** 1 The Rheumatism Research Center, Catholic Research Institute of Medical Science, The Catholic University of Korea, Seoul, South Korea; 2 Division of Rheumatology, Department of Internal Medicine, College of Medicine, The Catholic University of Korea, Seoul, South Korea; 3 Catholic Blood and Marrow Transplantation Center, College of Medicine, The Catholic University of Korea, Seoul, South Korea; 4 Transplant Research Center, Seoul St. Mary’s Hospital, College of Medicine, The Catholic University of Korea, Seoul, South Korea; Charité, Campus Benjamin Franklin, Germany

## Abstract

**Background:**

In this study we examined the *in vivo* and *in vitro* effects and mechanisms of action of curcumin on the development of acute graft-versus-host disease (GVHD) using a murine model.

**Methodology/Principal Findings:**

Mixed lymphocyte reactions were used to determine the *in vitro* effects of curcumin. Treatment with curcumin attenuated alloreactive T cell proliferation and inhibited the production of interferon (IFN)-γ and interleukin (IL)-17. In a murine acute GVHD model, transplantation of curcumin-treated allogeneic splenocytes into irradiated recipient mice significantly reduced the clinical severity scores of acute GVHD manifested in the liver, skin, colon and lung as compared with animals receiving vehicle-treated splenocytes. c-Fos and c-Jun expression levels in the skin and intestine, which are major target organs, were analyzed using immunohistochemical staining. Expression of both proteins was reduced in epithelial tissues of skin and intestine from curcumin-treated GVHD animals. The IFN-γ-expressing CD4^+^ splenocytes and IFN-γ-expressing lymph node cells were dramatically decreased in curcumin-treated mice. In contrast, CD4^+^Foxp3^+^ splenocytes were increased in the curcumin-treated acute GVHD animals. Flow cytometric analysis revealed that animals transplanted with curcumin-treated allogeneic splenocytes showed increased populations of CD4^+^ regulatory T cells (Tregs) as well as CD8^+^ Treg cells, compared to animals administered vehicle-treated splenocytes. Curcumin-treated acute GVHD animals could have a change in B cell subpopulations.

**Conclusion/Significance:**

In the present study, we investigated the efficacy and mechanism of action of curcumin treatment against acute GVHD. The acute GVHD mice administered with curcumin-treated splenocytes showed significantly reduced severity of acute GVHD. Curcumin exerted *in vivo* preventive effects on acute GVHD by reciprocal regulation of T helper 1 (Th1) and Treg (both CD4^+^ and CD8^+^ Treg) cell lineages as well as B cell homeostasis.

## Introduction

Allogenic hematopoietic stem cell transplantation (HSCT) is the only curative therapy with proven efficacy for the management of many hematologic malignancies and other life-threatening hematological diseases. However, the development of graft-versus-host disease (GVHD), which is the main complication of HSCT, is a significant obstacle of allogenic HSCT [1]. Acute GVHD mainly affects the skin, gastrointestinal tract, liver, and lung. The development of GVHD requires escalated and prolonged immunosuppressive therapy with increased risk of infectious complications. Ultimately, GVHD increases the risk of fatal morbidities and moralities in HSCT recipients. Although successive improvements in GVHD prevention have been achieved, complete protection from acute GVHD remains elusive. Acute GVHD (grades II–IV) occurs in 30–60% of patents after allogenic HSCT from human leukocyte antigen (HLA)-identical sibling donors [2]. Following the development of GVHD, complete remission has been observed in only 30 to 50% of patients with acute GVHD [3], [4].

Knowledge of the immunobiology underlying GVHD has advanced by virtue of immunology research in animal models, as well as clinical observations. GVHD occurs as a result of T cell activation followed by alloreactive T cell expansion and differentiation [5]. Acute GVHD is considered a process driven mainly by T helper 1 (Th1) and Th17 type immune responses. Th1 cell-associated cytokines involved in acute GVHD include interferon (IFN)-γ, interleukin (IL)-1, IL-6, and tumor necrosis factor (TNF)-α [6], [7]. Th17 cells are IL-17 producing T helper cells that are a lineage of CD4^+^ effector T cells distinct from the Th1 and Th2 cell lineages. Th17 cells were found to have a direct role in the development of GVHD [8]. Adoptive transfer of *in vitro*-differentiated Th17 cells is capable of inducing lethal acute GVHD [9]. Acting opposite of Th1 and Th17 cells, there are regulatory T (Treg) cells. Several observational studies have shown that a decreased population of circulating Treg cells was observed in patients that developed acute GVHD, compared to those without acute GVHD [10], [11]. Parallel to those findings, Treg cell expansion has been shown to be capable of reducing the severity of acute GVHD in murine acute GVHD models [12], [13]. Although there has been great advances in understanding the pathophysiology of GVHD, current GVHD prophylaxis and treatment are still based on non-specific immunosuppressive drug therapy [14].

Curcumin is a naturally occurring polyphenolic phytochemical that is derived from the root of the turmeric plant *Curcuna long*a and is responsible for the spicy taste of curries [15]. It is a nontoxic food that is used as a coloring agent and remains a vital ingredient of traditional medicine in India and China. More recently, antioxidant, anti-inflammatory, anti-microbial, and anti-carcinogenic properties of curcumin have been identified [16]–[20]. A small number of experiments revealed that curcumin treatment attenuated autoimmune diseases by downregulating IL-17 production [21] and shifting from Th1 to Th2 type responses [22]. However, its application in GVHD models has not been tested to date.

In the present study, we investigated the *in vivo* effect of curcumin in a murine model of acute GVHD. The acute GVHD model was developed by bone marrow transplantation, supplemented with varying numbers and different types of donor lymphocytes, into irradiated allogenic recipients that differ from the donors by major histocompatibility complex (MHC) class.

## Materials and Methods

### Mice

C57BL/6 (B6; H-2k^b^), and BALB/c (H-2k^d^) mice, 8–10 weeks old, were purchased from OrientBio (Sungnam, Korea). The mice were maintained under specific pathogen-free conditions in an animal facility with controlled humidity (55±5%), light (12 h/12 h light/dark), and temperature (22±1°C). The air in the facility was passed through a HEPA filter system designed to exclude bacteria and viruses. Animals were fed mouse chow and tap water *ad libitum*. The protocols used in this study were approved by the Animal Care and Use Committee of The Catholic University of Korea.

### BMT Model and Histopathology Scoring

Recipients (BALB/c) mice were injected intraveneously (i.v.) with 5×10^6^ total bone marrow cells from donor mice after lethal irradiation with 800 cGy. To induce acute GVHD, splenocytes were isolated from donor mice. Splenocytes (1×10^7^) from MHC major and minor antigen-disparate B6 donors were incubated with 20 nM cucurmin or control vehicle (DMSO) for 1 h at 37°C before adoptive transfer into recipient mice. To create GVHD–negative controls, 5×10^6^ T cell–depleted (TCD) bone marrow cells were transplanted into irradiated BALB/c recipient mice. All experiments were performed at least three times with six mice per group. Survival after BMT was monitored daily, and the degree of clinical GVHD was assessed using a scoring system that summed changes in five clinical parameters: weight loss, posture, activity, fir texture, and skin integrity. Mice were sacrificed on day 35 after BMT for blinded histopathological analysis of GVHD target tissues (skin, liver, and small and large intestine) [23]. Organs were harvested, cryo-embedded, and subsequently sectioned. Tissue sections were fixed in 10% buffered formalin and stained with hematoxylin and eosin (H&E) for histological examination.

### MLR Culture *in vitro*

Splenocytes derived from BALB/c mice were used as stimulator cells and those from B6 were used as responder cells in MLR assays. Spleen cells were removed using ammonium-chloride-potassium lysis buffer, washed, and resuspended in complete culture medium (RPMI 1640 medium supplemented with 10% heat-inactivated fetal calf serum, 1 mM sodium pyruvate, 5×10^5 ^M 2-ME, 20 mM HEPES, and antibiotics [100 U/mL penicillin, 100 ug/mL streptomycin]). Aliquots of 2×10^5^ CD4^+^ T cells (responders) were cultured with 2×10^5^ irradiated (2500 cGy) antigen-presenting cells (APC) in 96-well plates containing 200 μl of complete medium at 37°C in a humidified 5% CO_2_ atmosphere, pulsed with 1 µCi of [^3^H]-TdR (NEN Life Science Products Inc., Boston, MA, USA) for 18 h before harvesting, and counted using an automated harvester (PHD Cell Harvester; Cambridge Technology, Inc., Cambridge, MA, USA). Results are expressed as the mean counts per minute (cpm) of triplicate samples ± standard deviation (SD). The stimulation index was calculated by comparing the anti-stimulator response with the anti-self response.

### Flow Cytometry

Mononuclear cells were immunostained with various combinations of fluorescence-conjugated antibodies against CD4, CD25, Foxp3, IFN-γ, IL-4, IL-17, B220, IgD, IgM, CD95, GL-7, and streptavidin. These cells were also intracellularly stained with antibodies against IL-4 (BD Biosciences, San Jose, CA, USA), IL-10 (Biolegend, San Diego, CA, USA), IL-17, and Foxp3 (eBioscience, San Diego, CA, USA). Before intracellular staining, the cells were restimulated for 4 h with 25 ng/mL phorbol 12-myristate 13-acetate (PMA) and 250 ng/mL ionomycin in the presence of GolgiSTOP (BD Biosciences). Intracellular staining was conducted using an intracellular staining kit (eBioscience) according to the manufacturer’s protocol. Flow cytometric analysis was performed using a FACSCalibur cytometer (BD Biosciences).

### Measurement of Cytokines and IgG Titers

The concentrations of IFN-γ and IL-17 in cell culture supernatants and serum were measured using a sandwich ELISA (Duoset; R&D Systems, Lille, France). Serum levels of IgG, IgG1 and IgG2a antibodies were measured using a commercially available ELISA kit (Bethyl Laboratories, Montgomery, TX, USA).

### Immunohistochemistry

Immunohistochemistry was performed using the Vectastain ABC kit (Vector Laboratories, Burlingame, CA, USA). Tissues were first incubated with the primary anti-C-jun and anti-C-fos antibodies overnight at 4°C. The primary antibody was detected with a biotinylated secondary linking antibody, followed by incubation with a streptavidin–peroxidase complex for 1 h. The final color product was developed using DAB chromogen (DAKO, Carpinteria, CA, USA).

### Confocal Staining

Spleen tissue was obtained 14 days after BMT and was snap-frozen in liquid nitrogen and stored at −80°C. Tissue cryosections (7-µm thick) were fixed in 4% paraformaldehyde and stained using PE-labeled anti-IFN-γ, IL-4, IL-17, or Foxp3 antibody (eBioscience) and FITC-labeled anti-CD4 antibody and APC-labeled anti CD25 antibody (Biolegend). After incubation overnight at 4°C, stained sections were analyzed using a confocal microscope (LSM 510 Meta; Zeiss, Gottingen, Germany). CD4^+^IFN-γ^+^, CD4^+^IL-4^+^, CD4^+^IL-17^+^, CD4^+^CD25^+^Foxp3^+^ T cells were enumerated visually at higher magnification (projected on a screen) by four individuals.

### Statistical Analysis

Comparison of numerical data between three groups was performed with nonparametric Mann-Whitney tests. Statistical analysis was performed using SPSS 10.0 for Windows (SPSS, Chicago, IL, USA). *P* values <0.05 were considered significant. Data are presented as the mean ± SD.

## Results

### Curcumin Modulates Alloreative T Cell Responses *in vitro*

**Figure 1:**
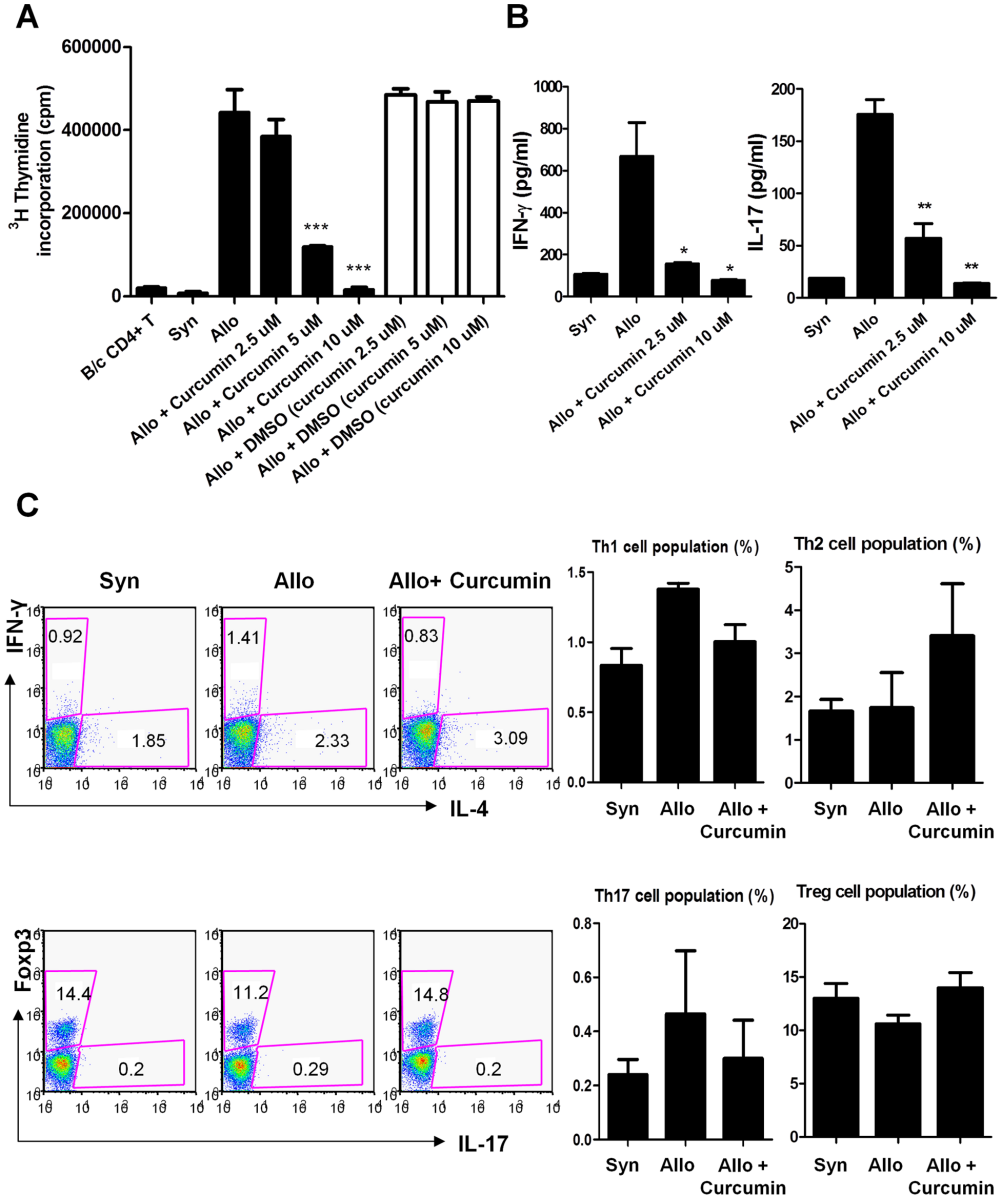
Curcumin inhibits alloreactive T cell responses and that is associated with downregulation of IFN-γ and IL-17. (**A**) A total of 10^5^ RBC-lysed C57BL/6 (B6) splenic T cells (responders) were incubated with 10^5^ irradiated RBC-lysed B6 splenic T cells (syngenic stimulators) or BALB/c splenic T cells (allogeneic stimulators) in an MLR. Curcumin was added on day 0, and T cell proliferation was measured by ^3^H-thymidine incorporation in each group. (**B**) The concentrations of IFN-γ and IL-17 in culture supernatants (A) were measured by ELISA. Data are shown as mean ± SD from at least 3 independent experiments. *P<0.05, **P<0.01, ***P<0.001. (**C**) A total of 10^5^ RBC-lysed B6 splenic T cells (responders) were incubated with 10^5^ irradiated and RBC-lysed B6 T cells (syngenic stimulators) or BALB/c splenic T cells (allogenic stimulators) in an MLR. Curcumin (2.5 µM) was added on day 0, and cells were harvested on day 4. Then, intracellular staining for IL-4, IFN-γ, IL-17, and Foxp3 in isolated CD4^+^ T cells was performed and analyzed by flow cytometric analysis. The flow cytometry data that are shown in (**C**) is representative of three independent experiments. Bars are shown as mean ± SD from at least 3 independent experiments.

To assess the effects of curcumin on the proliferative capacity of donor CD4^+^ T cells in response to alloantigens following transplantation, T cell alloreactivity was measured by [^3^H]-thymidine incorporation to assess T cell proliferation in mixed lymphocyte reactions (MLR). Proliferative responses to BALB/c T cells (allogeneic stimulator) was observed in C57BL/6 (B6) T cells (responder cells). Treatment with curcumin inhibited T cell alloreactivity in a dose-dependent manner (**Fig. 1A**). To ascertain whether or not the inhibitory effect on alloreactive T cell responses by curcumin was associated with the apoptosis induction. Annexin-V and propidium iodide (PI) double staining were performed and analyzed using flow cytometery (Fig. S1B). In addition, we performed MTT assays to determine whether curcumin treatment affects cell viability (Fig. S1A). The results indicated that the treatment with curcumin and DSMO at various concentrations did not induce apoptosis or affect cell viability. IFN-γ and IL-17 concentrations were then measured using an enzyme-linked immunosorbent assay (ELISA). Curcumin treatment significantly reduced IFN-γ and IL-17 level in culture supernatants (**Fig. 1B**). To determine whether *in vitro* curcumin treatment can regulate the Th1 and Th2 balance, B6 splenic T cells incubated with irradiated B6 splenic T cells (syngeneic stimulator) or BALB/c splenic T cells (allogeneic stimulator) in the absence of presence of curcumin (2.5 µM) were analyzed by intracellular staining for IL-4, IFN-γ, IL-17, and Foxp3 (**Fig. 1C**). While the Th1 cell population (IFN-γ^+^IL-4^−^ T cells) tended to decrease upon the curcumin treatment, the Th2 cell population (IFN-γ^−^IL-4^+^ T cells) tended to increase (statistically insignificant). Similarly, curcumin treatment generated reciprocal changes in Th17 and Treg cell differentiation, although the changes were not statistically significant (**Fig. 1C**).

### Transfer of Curcumin-treated Splenocytes Protects Mice from Acute GVHD after Bone Marrow Transplant

**Figure 2:**
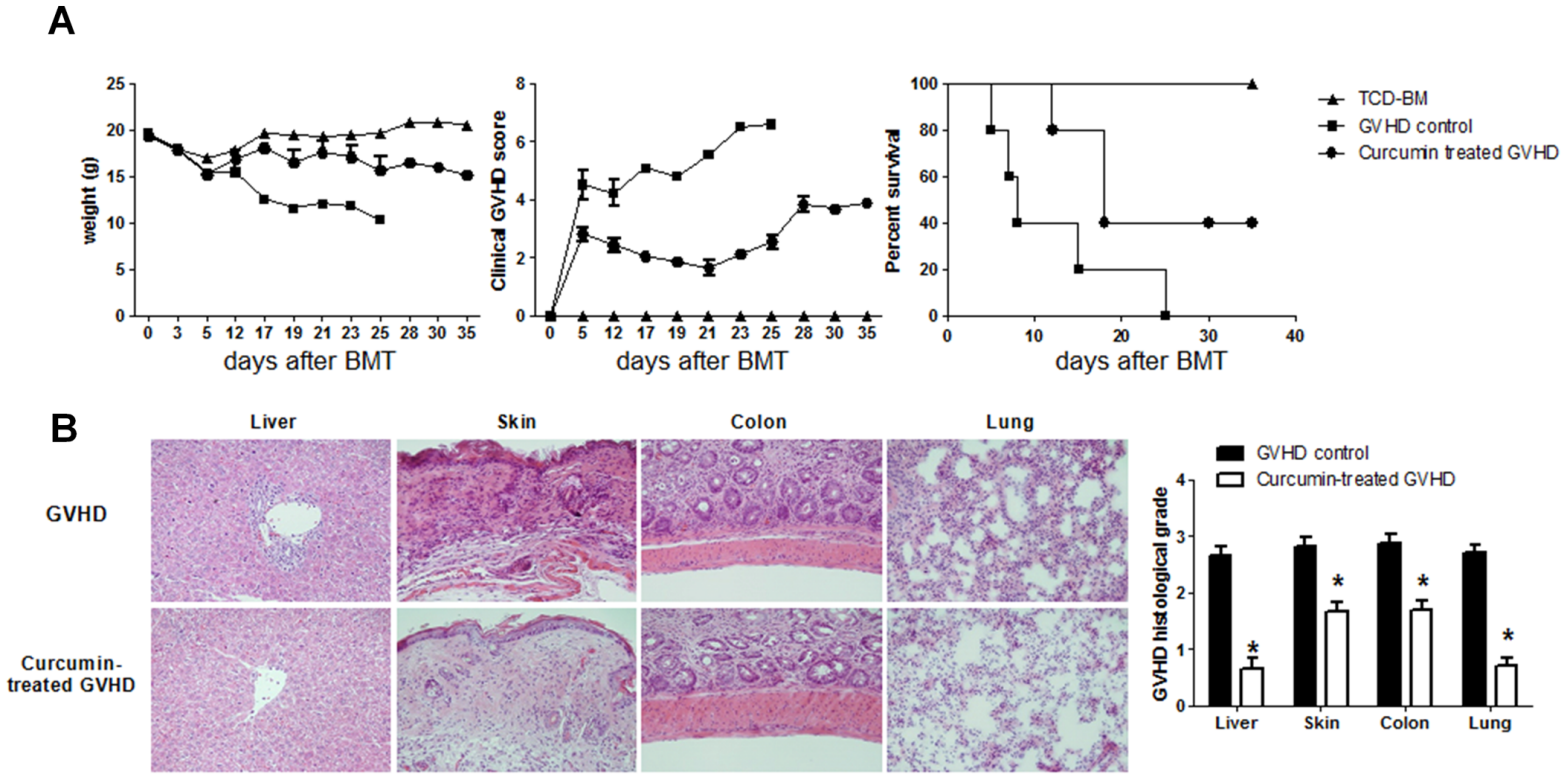
Blockade of AP-1 by curcumin reduces mortality from acute GVHD. (**A**) C57BL/6 (B6) splenocytes (1×10^7^ cells) were incubated with 10 µM curcumin or control vehicle (DMSO) for 1 h at 37°C before adoptive transfer into lethally irradiated (800 cGy) BALB/c (recipient) mice. Recipients also received 5×10^6^ total bone marrow cells from B6 mice and were monitored for weight loss, clinical signs of acute GVHD and recipients survival. Combined data from two independent experiments (n = 10 per group) are shown. (**B**) The left panels are representative tissue sections of liver, skin, colon and lung after transplantation of control (DMSO) or curcumin-treated (n = 6) splenocytes. Histology that are shown is representative of two independent experiments. This section was stained with H&E (original magnification, ×200). Right panels are average score of liver, skin, colon and lung of each group. Tissues were collected on day 14 after transplantation. Results are shown as mean ± SEM of 6 mice. *P<0.05.

**Figure 3:**
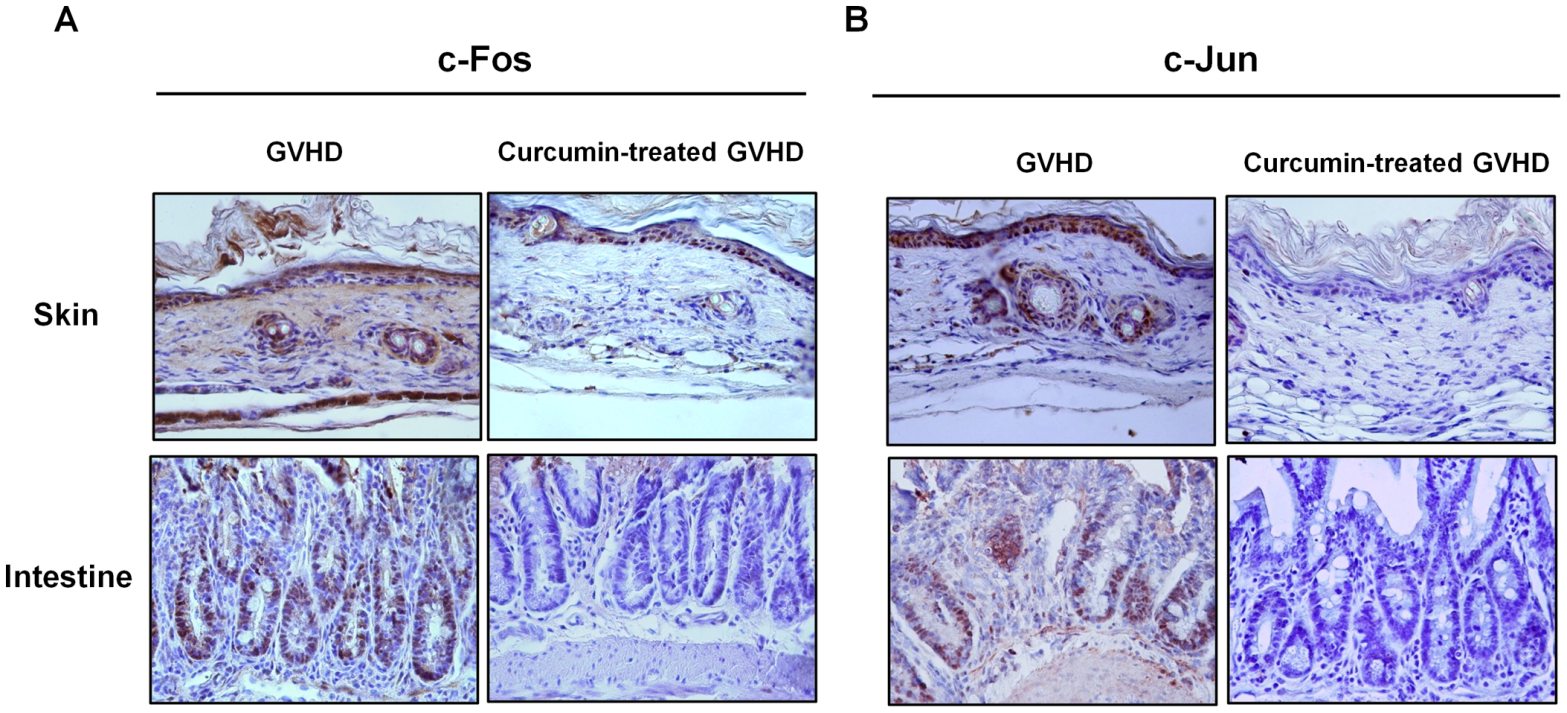
Reduced expression of c-Fos and c-Jun, components of AP-1, in skin and intestine from curcumin-treated GVHD animals. Representative examples of c-Fos (A) and c-Jun (B) immunohistochemical staining in skin and intestine tissue from GVHD mice. Positive immunoreactivity appears as a brown color and is counterstained with blue or green. Original magnification, ×400.

Because curcumin significantly suppressed alloreactivity *in vitro*, we next assessed its effects *in vivo* using a murine model of acute GVHD. Severe acute GVHD occurred in all BALB/c (H-2k^d^) recipient mice undergoing bone marrow transplantation (BMT) and infusion of donor (B6 mice, H-2k^b^) splenocytes that were cultured with or without curcumin (10 µM). Using this strategy, we found that acute GVHD animals transplanted with curcumin-treated splenocytes showed attenuated weight loss, less severe clinical scores of acute GVHD, and significantly delayed acute GVHD lethality in recipient mice, compared to the vehicle-treated group (**Fig. 2A**). Liver, skin, colon and lung are the target organs of acute GVHD. Mice from each treatment group were sacrificed on day 14 post-BMT after GVHD induction. To determine the protective effects of curcumin on the development of acute GVHD, we evaluated tissue pathology in liver, skin, colon and lung. As shown in Fig. 2B, moderate to severe acute GVHD was noted in those organs of vehicle-treated animals. Curcumin treatment on donor splenocytes significantly improved pathologic severity scores in the liver, skin, colon and lung. To ascertain whether the protective functions of curcumin work by inhibiting the function of nuclear activator protein-1 (AP-1), the expression of its components, c-Fos and c-Jun, in the skin and intestine were assessed by immunohistochemical staining. Expression of both proteins in epithelial tissues of skin and intestine was significantly reduced in curcumin-treated acute GVHD animals (**Fig. 3A, B**).

### Curcumin Acts in the Acute GVHD Model via Reciprocal Regulation of Th1 and Treg Cells

**Figure 4:**
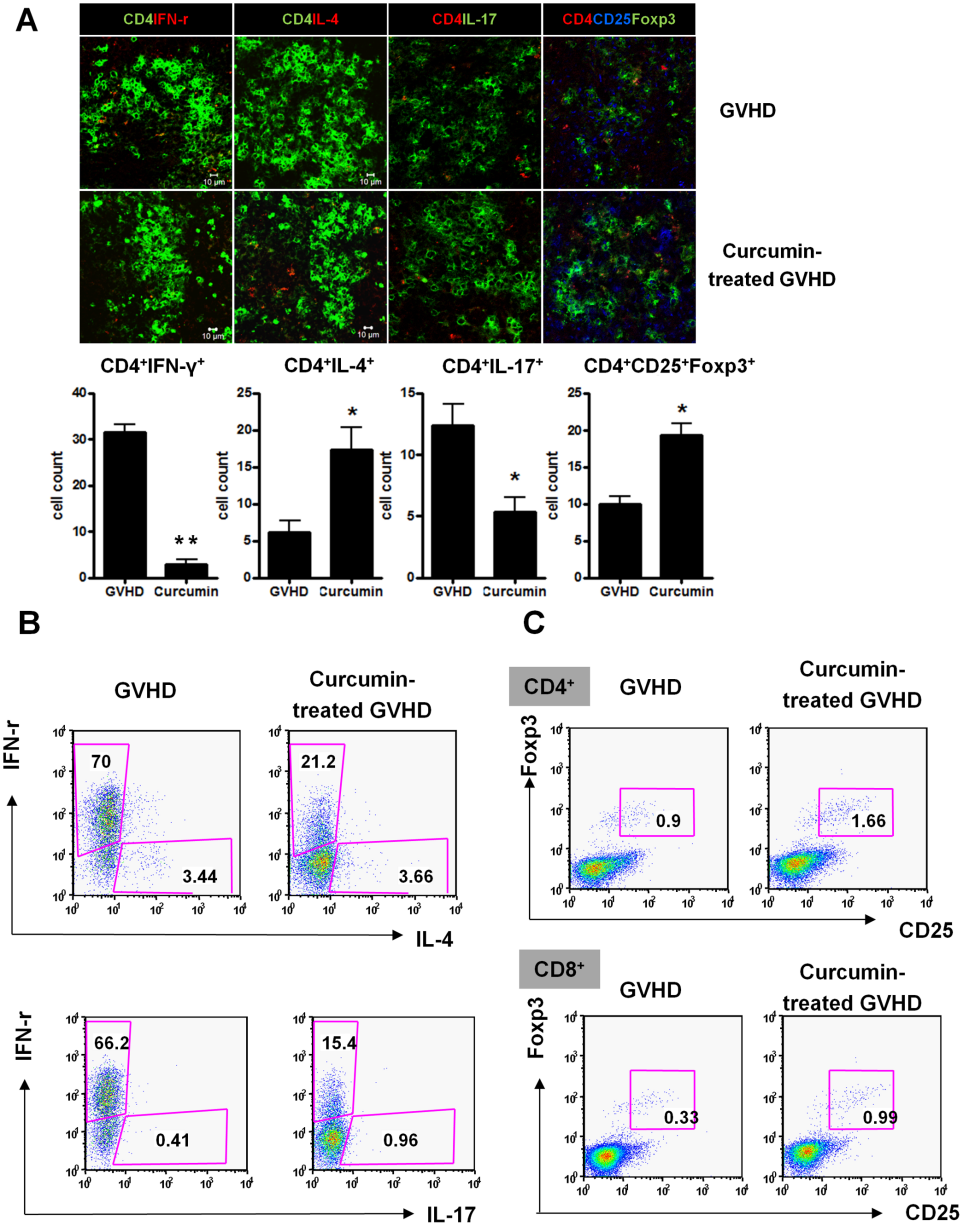
Analysis of CD4^+^ T helper cells in curcumin-treated GVHD mice. (**A**) C57BL/6 (B6) splenocytes (1×10^7^ cells) were incubated with 10 µM curcumin or control vehicle (DMSO) for 1 h at 37°C before adoptive transfer into lethally irradiated (800 cGy) BALB/c mice. Recipient BALB/c mice also received 5×10^6^ total bone marrow cells from B6 mice. Intracellular cytokines were determined in the splenocytes of each group and were analyzed by confocal microscopy on day 14 after BMT. CD4^+^IFN-γ^+^, CD4^+^IL-4^+^, CD4^+^IL-17^+^, CD4^+^CD25^+^Foxp3^+^ T cells were enumerated visually at higher magnification (projected on a screen) by four individuals, the mean values are presented in the form of a histogram. *P<0.05, **p<0.001 *versus* the vehicle-treated group. Results are shown as mean ± SD (n = 5 mice per group). (**B**) Fourteen days after BMT, lymph node cells were isolated from each group and then analyzed by flow cytometry for the expression of IL-4, IL-17, and IFN-γ. The experiment was performed once with six mice per group. (**C**) Fourteen days after BMT, splenocytes isolated from each group were stained with anti-CD4 and anti-CD8 antibodies followed by intracellular IFN-γ, IL-4, Foxp3, and IL-17 antibodies and examined by flow cytometry. The data is representative of at least three independent experiments.

To investigate the *in vivo* mechanism of curcumin in the acute GVHD murine model, the numbers of CD4^+^IFN-γ^+^, CD4^+^IL-4^+^, CD4^+^IL-17^+^, and CD4^+^CD25^+^Foxp3^+^ T cells in spleens isolated from each group were counted using confocal staining. The numbers of CD4^+^IFN-γ^+^ and CD4^+^IL-17^+^ T cells in spleens were decreased in curcumin-treated GVHD animals as compared with the vehicle-treated group. On the other hand, the numbers of CD4^+^IL-4^+^ and CD4^+^CD25^+^Foxp3^+^ splenocytes were increased in the curcumin-treated group, although the differences between the two groups were modest (**Fig. 4A**). Fourteen days after BMT, lymph node cells isolated from each group were analyzed for the expression of IL-4, IL-17, and IFN-γ. The number of lymph node cells expressing IFN-γ was significantly decreased in the curcumin-treated group, whereas IL-4- and IL-17-expressing lymph node cells did not decrease, but rather increased moderately (**Fig. 4B**). To determine whether treatment with curcumin affected the number of circulating regulatory T cells *in vivo*, CD4^+^ and CD8^+^ splenocytes isolated from each group were analyzed for CD25 and Foxp3 expression. Interestingly, the CD25^+^Foxp3^+^ subset of CD4^+^ splenocytes was increased upon treatment with curcumin. In addition, the percentage of CD8^+^CD25^+^Foxp3^+^ cells also increased in the curcumin-treated group (**Fig. 4C**). Taken together, curcumin treatment on donor splenocytes significantly inhibited IFN-γ expression and reciprocally expanded Treg population in recipient mice. On the other hand, the present study showed that effects of curcumin on IL-4 and IL-17 expressions demonstrated conflicting results in splenocytes and lymph node cells. Next, we determined to identify whether or not curcumin treatment affects cell populations of hematopoietic stem cell (HSC), dendritic cell (DC), and natural killer cells (NK cells) in recipient mice after BMT. There was no significant difference in HSC (c-kit or CD34-expressing cells) and DC (CD11c-expressing cells) population in spleens and bone marrows that were isolated from mice of each group. Although the difference was marginal, NK cell (NK1.1-expressing cells) population in spleens and bone marrows tended to decrease in curcumin-treated group, compared to that of vehicle-treated mice (Fig. S2). Based on the flow cytometry data that showed the majority of CD4^+^ T cells and whole splenocytes expressed H-2k^b^ but not H-2k^d^, those cells from mice transplanted with curcumin- and vehicle-treated splenocytes almost originated from donor cells. And curcumin treatment on donor splenocytes did not affect absolute number of T cell subsets in recipient mice (Fig. S3). Conclusively, the attenuated severity of acute GVHD following transplantation with curcumin-treated splenocytes may result from the restoring balance between Th1, and Treg differentiation, not through the alteration of absolute number of immune cells such as T cells, HSC, DC, and NK cells.

### Curcumin Treatment Altered B Cell Subpopulations

**Figure 5:**
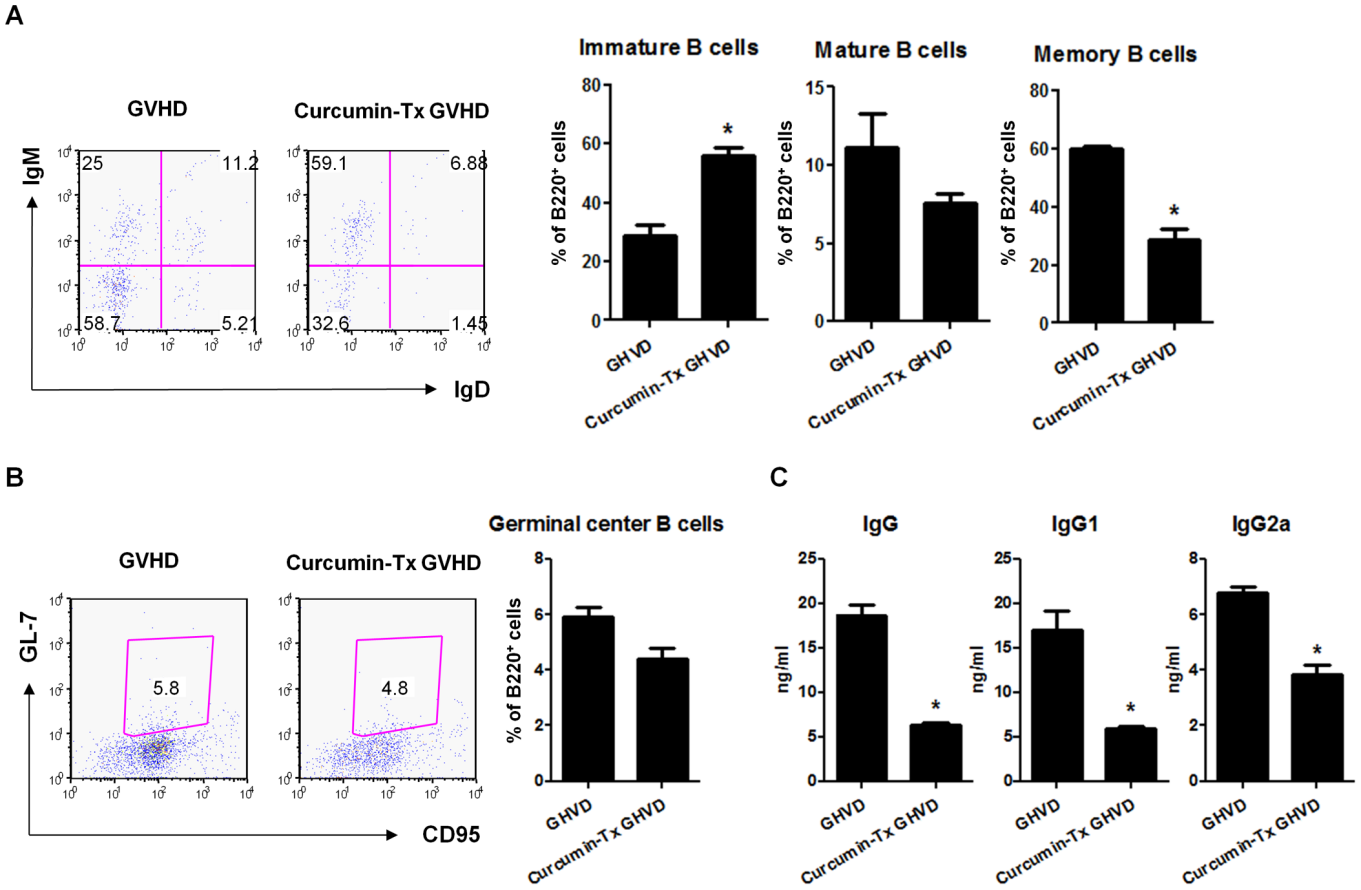
Analysis of B cell subsets in spleens of GVHD mice. C57BL/6 (B6) splenocytes (1×10^7^ cells) were incubated with curcumin (10 µM) or vehicle control (DMSO) for 1 h at 37°C before adoptive transfer into lethally irradiated (800 cGy) BALB/c mice. Recipients also received 5×10^6^ total bone marrow cells from B6 mice. (**A and B**) On day 14 after BMT, B cell subsets were analyzed. Splenocytes isolated from vehicle or curcumin–treated GVHD mice were stained for B220, IgM, and IgD (**A**) or B220, CD95, and GL-7 (**B**) and then analyzed by flow cytometry. Cells shown were gated on B220. Numbers indicate the percent of each B cell subtypes in each outlined area. B220^+^ B cells included IgM^high^IgD^low^ (immature B cells), IgM^high^IgD^high^ (mature B cells), and IgM^low^IgD^low^ (memory B cells). The proportion of germinal center B cells within B220^+^ cells in the spleen decreased in the curcumin-treated group (**B**). The mean concentrations of serum IgG and IgG1 determined by ELISA were lower in curcumin-treated mice as compared with those of the vehicle-treated group (**C**). Values are showed as mean ± SEM (n = 3 animals per group). For A and B, one representative experiment of three independent experiments is shown.

To determine whether there was a change in B cell subpopulations due to curcumin treatment, splenocytes from acute GVHD mice were analyzed. Flow cytometric analysis revealed that the immature B cell portion among B220^+^ B cells was increased in curcumin–treated acute GVHD animals, whereas the mature B cell and memory B cell subsets were decreased (**Fig. 5A**). Similarly, the proportion of GL-7^+^CD95^+^ germinal center B cells was decreased in the curcumin–treated group (**Fig. 5B**). The absolute numbers of B cell subpopulations also showed similar trend (Fig. S4). The mean concentrations of IgG, IgGa and IgG2a, respectively, were decreased in the sera of curcumin-treated animals as compared with those of the vehicle-treated group (**Fig. 5C**).

## Discussion

Curcumin, which is the orange-yellow component of curry powder, is a natural polyphenol product with anti-inflammatory and anti-cancer properties [24]. There have been various studies that have shown the anti-cancer, anti-viral, and anti-inflammatory properties of the compound [25]–[27]. This is the first study to investigate the *in vivo* and *in vitro* effects of curcumin on the severity of acute GVHD. *In vitro*, curcumin inhibited alloreactive T cell proliferation and Th1 cell lineage differentiation. *In vivo*, mice that received curcumin–treated splenocytes showed diminished severity scores of acute GVHD, and this inhibition of acute GVHD by curcumin was associated with inhibition of the AP-1 signaling pathway. Surprisingly, transplantation with curcumin–treated allogenic splenocytes (allogenic stimulator) resulted in increased populations of CD4^+^ Treg cells, as well as CD8^+^ Treg cells in recipient mice, compared to those of mice transplanted with vehicle-treated splenocytes.

Along with HLA incompatibility, the intensity of conditioning therapy is known to be a risk factor for the development of acute GVHD [28]. Unfortunately, acute GVHD does develop despite the administration of prophylactic agents, such as calcineurin inhibitors and methotrexate. Upon the occurrence of acute GVHD after HSCT, many patients should take immunosuppressive agents despite the increased risk of severe infection and many other adverse events. Our present study suggests the therapeutic potential of curcumin, which has been used safely for a long time.

Although the pathophysiology of acute GVHD is complex, it develops due to donor T cell responses to host alloantigens expressed by host antigen-presenting cells and subsequent dysregulation of inflammatory cytokine cascades [5], [29]. Classically, acute GVHD is also considered to be predominantly related to Th1 responses [30]. However, recent scientific investigations have discovered the possible role of Treg and Th17 cells in the development of GVHD [31]. The results obtained from our present study correspond with other previous reports that showed a shift from Th1 to Th2 responses by curcumin and its reciprocal effects on Th17/Treg cells [21], [32], [33]. In the current study, the increased populations of CD8^+^ Treg cells along with CD4^+^ Treg cells by curcumin treatment were associated with attenuated acute GVHD severity in a murine model. The novelty of our study was the finding of increased CD8^+^ Treg cells by curcumin treatment.

Treg cells are known to have suppressive effects on autoreactive lymphocytes and to control innate and adaptive immunity [34]. Removal of Treg cells from the donor graft dramatically accelerated GVHD in an experimental GVHD model [35]. Conversely, ongoing GVHD was ameliorated by infusion with donor or host Treg cells [36], [37]. Although the beneficial effects of Tregs in human GVHD were uncertain up until now, the finding that peripheral blood from patients with GVHD demonstrated reduced numbers of Foxp3^+^CD4^+^CD25^+^ T cells suggested the potential benefits of the clinical application of Treg cells [38], [39]. Accumulating evidence from experimental animal studies suggest that the adoptive transfer of Tregs is a potential strategy to suppress or prevent human GVHD. However, the relative scarcity of circulating Tregs and the difficulty in isolating pure Treg cells remain critical obstacles to carrying out this promising strategy. If curcumin induces the expansion of the Treg population in humans, the compound could be an adjunctive therapy in allogenic HSCT. However, there are controversies that surround the effects of curcumin on the number and immunomodulatory function of Treg cells. Zhao *et al*. recently showed that curcumin inhibits the immunosuppressive activity of Treg cells *in vitro* [40]. In that study, Foxp3, a critical regulator of Treg cell development and function, was downregulated by curcumin treatment. Conversely, one recent study revealed the induced differentiation of the Treg lineage by curcumin-treated dendritic cells [33].

Curcumin was revealed to enact its immunomodulatory effect through the inhibition of several transcriptional factors, including AP-1 signaling [41]. In the present study, the inhibitory effect on acute GVHD by curcumin was associated with attenuated AP-1 activity in skin and intestine. Skin and gut epithelial tissues induce class II HLA, consequently promoting specific targeting during acute GVHD [42], [43]. Skin keratinocytes expressing endogenous tissue antigens can directly prime naïve T cells [44], contributing the development of skin GVHD. In gut GVHD, intestinal epithelial cells are preferential target cells damaged by infiltrating donor T lymphocytes [45]. In our present study, the inhibitory effects of curcumin on the development of GVHD were associated with attenuated expressions of c-Fos/c-Jun in the epithelial tissues of skin (including keratinocytes) and intestine, suggesting that decreased AP-1 signaling in skin keratinocytes and intestinal epithelial cells may at least contribute to the attenuated severity of acute GVHD in animal models. Within our knowledge, little is known about the pathobiological roles of AP-1 signaling in GVHD development.

Although acute GVHD is considered to be mediated mainly by donor T cells, recent animal studies suggest that B cells might also play an important role in the biology of GVHD [46]. The specific mechanisms of B cells involved in the development of acute GVHD remains in large part unknown until now. Nevertheless, some circumstantial evidence for the pathological role of B cells in acute GVHD comes from clinical reports demonstrating that anti-B cell therapy such as rituximab reduced the incidence and severity of acute GVHD [47]. In line with previous studies, our study also showed that the preventive effect of curcumin on acute GVHD might be associated with a change of B cell homeostasis.

In the present study, curcumin-treatment on donor splenocytes did not affect the absolute number of CD4^+^, CD8^+^ T cells, B cells, and other immune cells including NK cells, HSCs, and DCs in recipients. Our study showed that the inhibitory effects of curcumin on the development of acute GVHD after BMT were associated with altered subset of T and B cells, not associated with absolute number of immune cells. On the other hand, previous studies that identified anti-cancer effects of curcumin have showed its beneficial effect was achieved through induction of T cell apoptosis even in normal T cells *via* increasing endoplasmic reticulum stress [48]–[50]. Although there is less research on anticancer effect of curcumin on B cells, curcumin can selectively induce apoptosis of B lymphoma cells [51] and showed anti-inflammatory effects through repressing B call-activating factor belonging to the TNF family [52], [53]. To our knowledge, this is the first study that shows immunomodulatory effects of curcumin *in vivo* treatment through the altered subpopulations of B cells, not affecting cell viability.

In conclusion, the present report showed that curcumin inhibited alloreactive T cell responses and IFN-γ and IL-17 production *in vitro*. Transplantation of curcumin-treated splenocytes attenuated acute GVHD severity and shifted responses from Th1 to Th2 *in vivo*. Interestingly, transplantation with curcumin-treated splenocytes resulted in the expansion of CD4^+^ as well as CD8^+^ Tregs *in vivo*. Thus, our present observations reveal a promising strategy to prevent lethal acute GVHD through the expansion of Treg cells. Recipient mice transplanted with curcumin-treated splenocytes showed altered B-cell subpopulation, increased immature B cells and reciprocally decreased mature- and memory B cells, compared with those transplanted with vehicle-treated splenocytes.

## Supporting Information

Figure S1 **The inhibitory effect of curcumin on alloreactive T cell responses is not associated with apoptosis induction or decreased cell viability.** (A) Cell apoptosis analyzed by flow cytometry. The lower left Annexin-V−/propidium iodide (PI)– represents normal healthy cells. The lower right Annexin-V+/PI– and upper right Annxin-V+/PI+ quadrant represent early and later apoptotic cells, respectively. The upper left quadrant, Annexin-V−/PI+ represent necrotic cells. (B) Cell viability as evaluated with the MTT assay. Values of MTT assay on cell viability after the different treatment with curcumin or DMSO (diluent). Bars are shown as means ± SEM from at least 3 independent experiments.
(TIF)

Figure S2 **Effect of hematopoietic stem cell and other immune cell by curcumin.** (A) CD34- or c-Kit-expressing hematopoietic stem cell, (B) CD11c-expressing dendritic cells, and (C) NK1.1-expressing natural killer cell populations among splenocytes and bone marrow cells were analyzed by flow cytomertry.
(TIF)

Figure S3 **Analysis of immune reconstitution after BMT.** (A) Splenocytes and CD4+ T cells of BMT mice tranaplanted with vehicle- and curcumin-treated splenocytes originate from donor cells expressing H-2k^b^. (B) Absolute number of CD4^+^ and CD8^+^ T cells were similar between mice transplanted with vehicle- and curcumin-treated splenocytes.
(TIF)

Figure S4 **Analysis of B cell subset after BMT.** Absolute number of B cell subpopulation among B220^+^ B cells were shown in BMT mice and were compared between vehicle- and curcumin-treated groups.
(TIF)
