# Supplementary figures and images for: Curcumin Attenuates Acute Graft-versus-Host Disease Severity via In Vivo Regulations on Th1, Th17 and Regulatory T Cells
Source: PLoS One. 2013 Jun 20;8(6):e67171. doi: 10.1371/journal.pone.0067171 (PMC3688629; doi:10.1371/journal.pone.0067171)

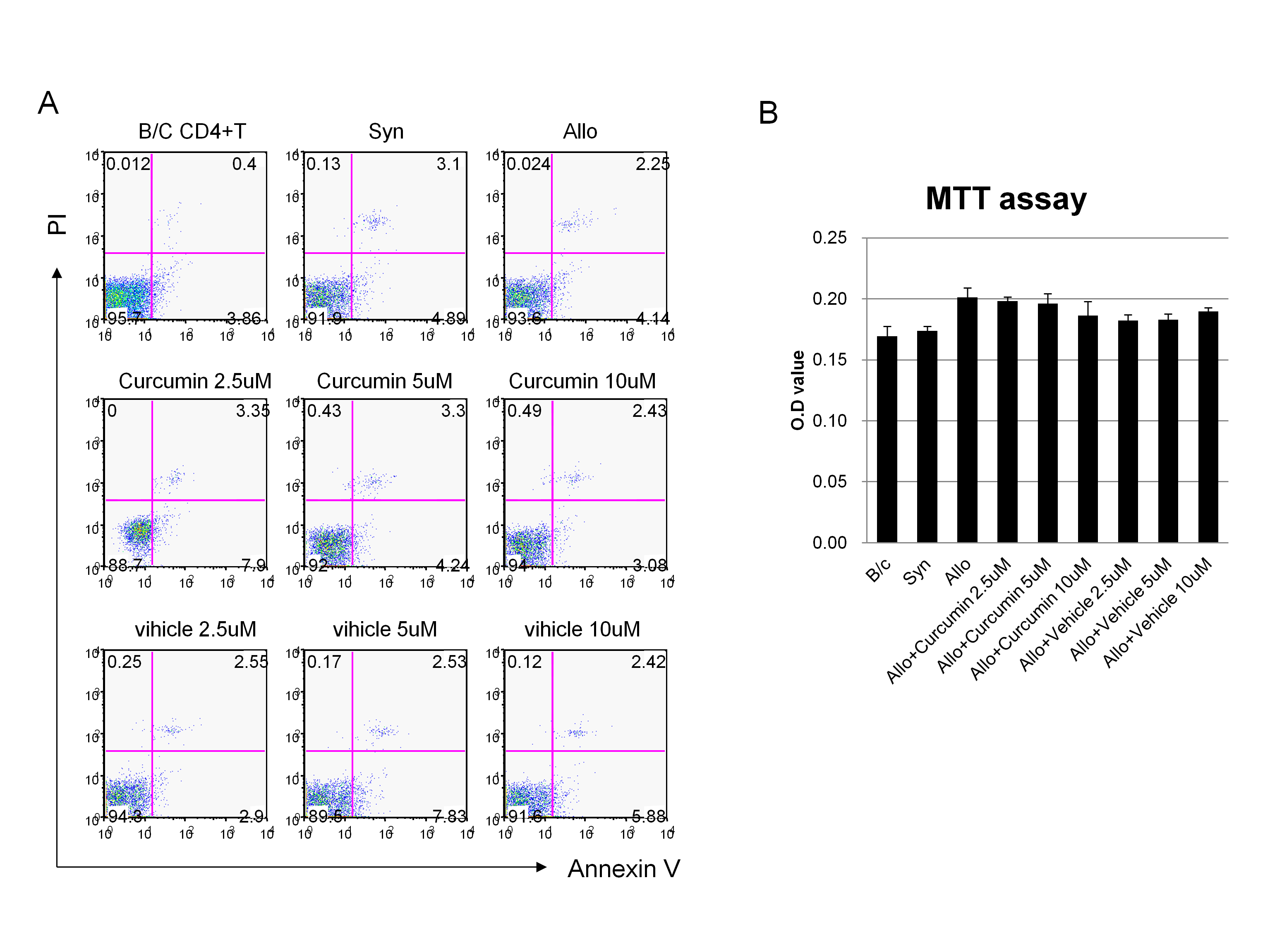

Supplement: Figure S1 — The inhibitory effect of curcumin on alloreactive T cell responses is not associated with apoptosis induction or decreased cell viability. (A) Cell apoptosis analyzed by flow cytometry. The lower left Annexin-V−/propidium iodide (PI)– represents normal healthy cells. The lower right Annexin-V+/PI– and upper right Annxin-V+/PI+ quadrant represent early and later apoptotic cells, respectively. The upper left quadrant, Annexin-V−/PI+ represent necrotic cells. (B) Cell viability as evaluated with the MTT assay. Values of MTT assay on cell viability after the different treatment with curcumin or DMSO (diluent). Bars are shown as means ± SEM from at least 3 independent experiments. (TIF) [file pone.0067171.s001.tif]

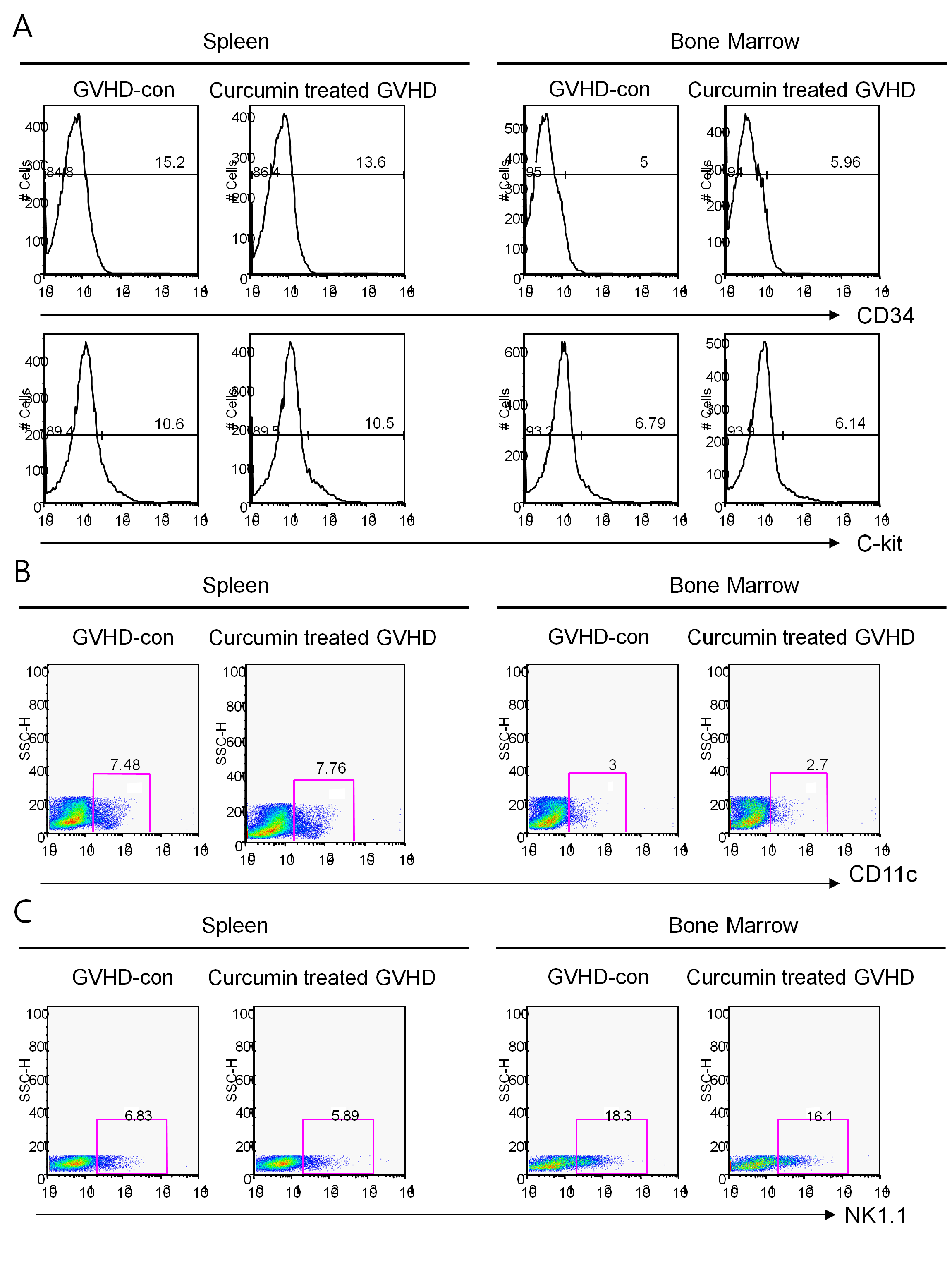

Supplement: Figure S2 — Effect of hematopoietic stem cell and other immune cell by curcumin. (A) CD34- or c-Kit-expressing hematopoietic stem cell, (B) CD11c-expressing dendritic cells, and (C) NK1.1-expressing natural killer cell populations among splenocytes and bone marrow cells were analyzed by flow cytomertry. (TIF) [file pone.0067171.s002.tif]

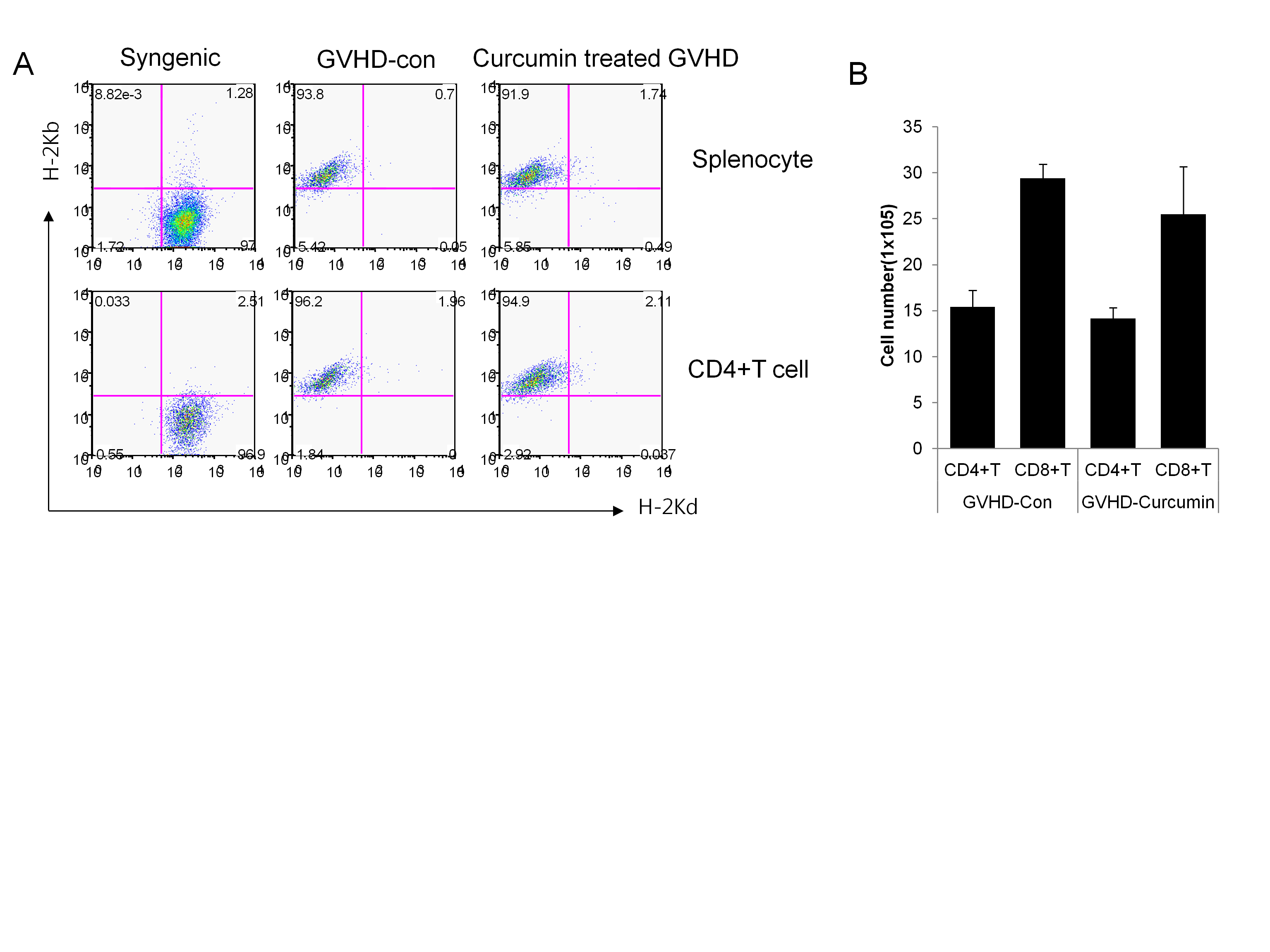

Supplement: Figure S3 — Analysis of immune reconstitution after BMT. (A) Splenocytes and CD4+ T cells of BMT mice tranaplanted with vehicle- and curcumin-treated splenocytes originate from donor cells expressing H-2kb. (B) Absolute number of CD4+ and CD8+ T cells were similar between mice transplanted with vehicle- and curcumin-treated splenocytes. (TIF) [file pone.0067171.s003.tif]

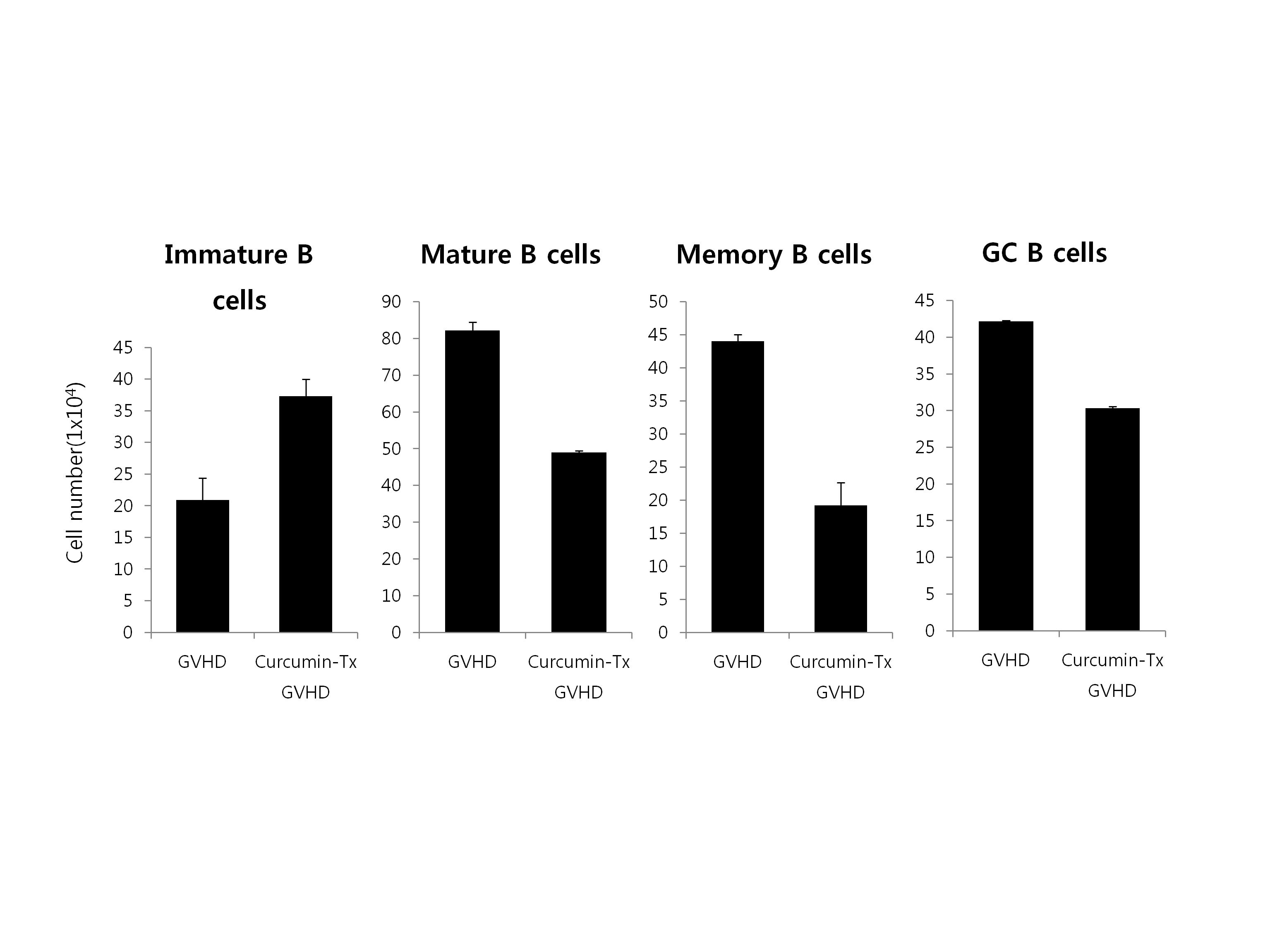

Supplement: Figure S4 — Analysis of B cell subset after BMT. Absolute number of B cell subpopulation among B220+ B cells were shown in BMT mice and were compared between vehicle- and curcumin-treated groups. (TIF) [file pone.0067171.s004.tif]
